# Efficacy of zinc pyrithione preservative incorporated into a female external urine wicking device in reducing growth of healthcare-associated pathogens: a laboratory evaluation

**DOI:** 10.1017/ash.2026.10866

**Published:** 2026-09-21

**Authors:** Jennifer L. Cadnum, Samir Memic, Curtis Donskey

**Affiliations:** 1 VA Northeast Ohio Healthcare System, USA; 2 VA Northeast Ohio Health Care: VA Northeast Ohio Healthcare System, USA; 3 Infectious Diseases Section, Louis Stokes Cleveland VA Medical Center, Cleveland, USA

## Abstract

Female external urine wicking devices (FEUWDs) are an alternative to indwelling urinary catheters that may decrease the risk of urinary tract infection. We found that sponge sections from a commercial FEUWD impregnated with zinc pyrithione and thiabendazole inhibited growth of bacterial and fungal pathogens, whereas 3 other FEUWDs did not.

## Introduction

Female external urine wicking devices (FEUWDs) are increasingly used as an alternative to indwelling urinary catheters.^1^ These devices avoid the presence of foreign bodies within the urethra and bladder and may decrease catheter-associated urinary tract infections. In a systematic review and meta-analysis, implementation of FEUWDs was associated with a nonsignificant 32% reduction in indwelling CAUTI rates, with a significant 54% reduction when analysis was limited to studies describing protocols for FEUWD implementation.^1^ Inconsistent reporting made it impossible to assess the rates of FEUWD-associated UTI.^1^

Although FEUWDs may pose a relatively low risk for UTI, they could potentially serve as a nidus for overgrowth of urinary pathogens in proximity to the urinary system where they could ascend into the bladder. Exchange of the devices is recommended immediately if soiled with feces or blood or every 8 to 12 hours or 12 to 24 hours. However, one manufacturer recommends exchange after up to 48 hours and has incorporated thiabendazole and zinc pyrithione into the device to inhibit microbial growth. Thiabendazole has antifungal activity against dermatophytes, but not *Candida albicans*.^2^ Zinc pyrithione has activity against many bacteria and fungi and is commonly used in skincare and haircare products.^3^ Zinc pyrithione provides persistent activity on skin attributed to adherence to hair and the stratum corneum.^3^ Incorporation of zinc pyrithione into cellulose sponges or fabrics provides antimicrobial activity that persists after rinsing washing.^4,5^ Here, we tested whether the FEUWD containing zinc pyrithione and thiabendazole inhibits growth of microbes.

## Methods

### FEUWDs tested

The CareDry Female External Catheter (Boehringer Laboratories, Phoenixville, PA) has thiabendazole and zinc pyrithione incorporated into the sponge material. The other FEUWDs, which did not contain inhibitory agents, included PureWick System (PureWick, El Cajon, CA), Versette External Urinary Catheter (Medline Industries, LP, Northfield, IL), and Sage PrimaFit External Urine Management (Sage Products, LLC, Carey, IL).

### Test microbes and susceptibility testing

The test microbes included a clinical methicillin-resistant *Staphylococcus aureus* (MRSA) isolate, *Candidozyma auris (*previously *Candida auris)* Antibiotic Resistance Bank (AR)-0385 (clade IV), *Candida albicans* American Type Culture Collection (ATCC) strain 10231, a clinical carbapenem-resistant *Klebsiella pneumoniae* strain (VA367), and carbapenem-resistant *Escherichia coli* ATCC strain BAA-2340. Agar dilution minimum inhibitory concentrations of thiabendazole (Thermo Fisher Scientific, Waltham, MA) and zinc pyrithione (Millipore Sigma, Burlington, MA) for the test microbes were determined using standard methods for susceptibility testing of bacteria and *Candida* species.^6^

### Growth of the test microbes in nutrient broth containing FEUWD sponge material

For each FEUWD, 1 cm^2^ sections of sponge material that wicks urine were submerged in 5 mL of trypticase soy broth (TSB) (Sigma-Aldrich, Burlington, MA). Ten µL containing 10^6^ to 10^7^ colony-forming units (CFU) of the test microbes in phosphate-buffered saline (PBS) were added and vortexed followed by incubation at 37°C. TSB with no sponge material was used as a control. Aliquots obtained at time 0, 2, 6, 24, and 48 hours were diluted 1:100 v/v in PBS; preliminary testing demonstrated neutralization of antimicrobial activity with 1:100 v/v dilution.^7^ Dilutions were plated on selective media to quantify the microbes.

### Persistence of antimicrobial activity in the FEUWD with preservatives

For 5 CareDry FEUWDs, we tested antimicrobial activity against the MRSA isolate after up to 3 liters of artificial urine had flowed through the device. Artificial urine was prepared as described previously.^8^ The FEUWD was placed in a container holding 0, 0.1, 1, 2, or 3 L of artificial urine. Vacuum suction was turned on to pull all the urine through the sponge material into the catheter and then to a suction canister. After passage of urine, 1 cm^2^ sections were cut from the sponge material and assessed for inhibition of growth of MRSA in TSB as described previously. TSB with no sponge was used as a control. Aliquots were obtained at time 0, 24, 48, and 120 hours to quantify MRSA.

### Data analysis

A linear model with adjustment for time and microbe type was used to compare concentrations of each of the test organisms grown in TSB versus in TSB containing FEUWD sponge material. Analyses were performed in R version 4.2.2 (R Foundation for Statistical Computing, Vienna, Austria).

## Results

### Susceptibility of the test microbes to zinc pyrithione and thiabendazole

The MIC of thiabendazole for each of the test organisms was >128 µg/mL. The MIC of zinc pyrithione for the *C. auris*, *C. albicans*, *K. pneumoniae*, and *E. coli* test strains was 4 µg/mL; the MIC for the MRSA test strain was 2 µg/mL.

### Growth of the test microbes in nutrient broth containing FEUWD sponge material

**Figure 1.**
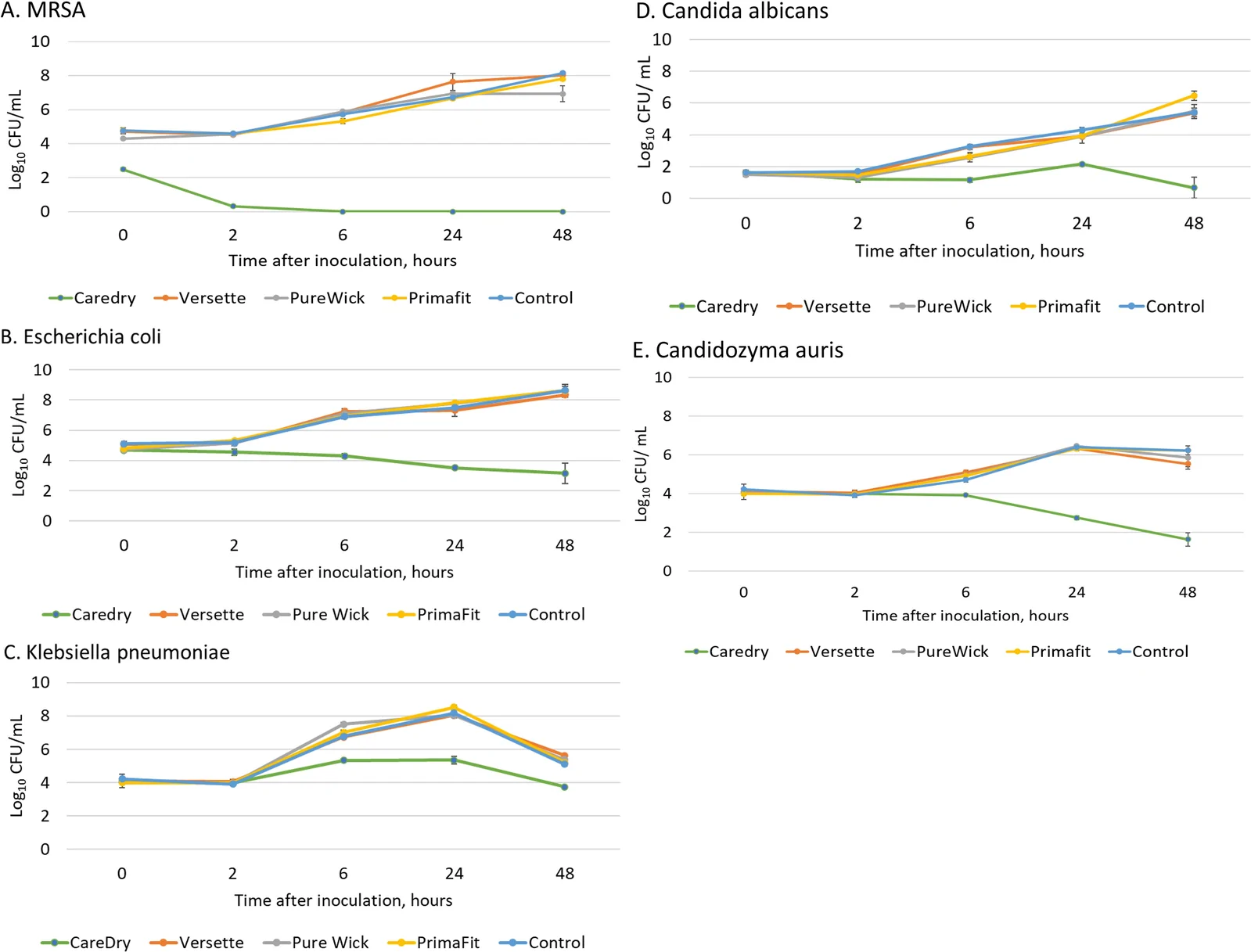
Figure 1 long description. Growth of test microbes in nutrient broth containing sponge material from female external urinary wicking devices. The control was trypticase soy broth with no sponge material. Error bars show standard error.

Figure 1 shows the shows the growth curves for the test microbes. The concentration of the test organisms increased by 2 to 4 log_10_ after 24 to 48 hours when incubated in TSB. For the CareDry FEUWD, the concentration of all the test microbes was significantly reduced in comparison to TSB (*P* ≤.001); MRSA decreased to undetectable levels by 6 hours and *C. auris* decreased by ∼2 log_10_ after 24 to 48 hours, whereas the levels of the other microbes remained at the level of the initial inoculum. For the other FEUWDs, growth of the microbes did not differ from growth in TSB (*P* ≥.9).

### Persistence of antimicrobial activity in the FEUWD with preservatives

**Figure 2.**
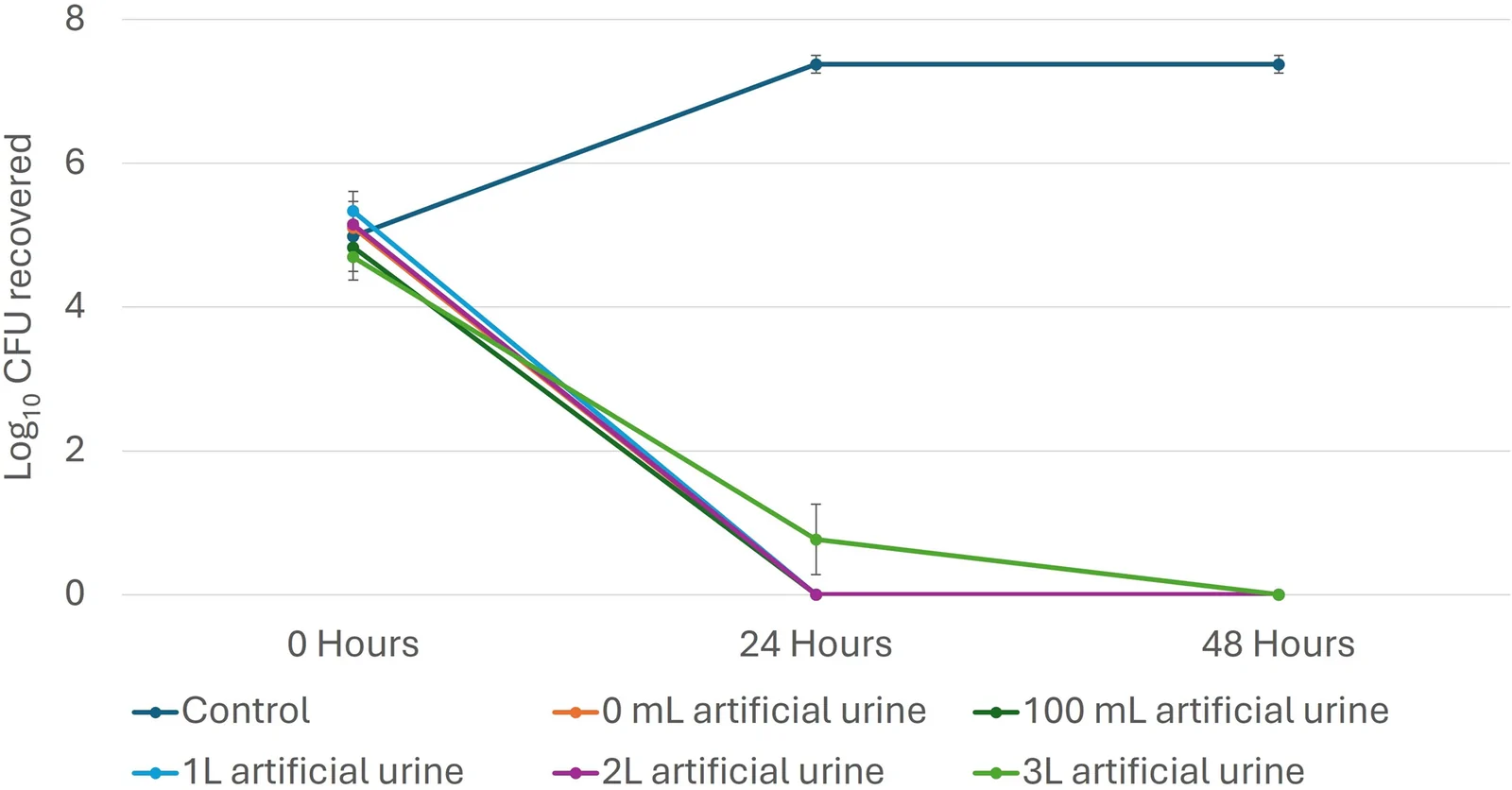
Growth of methicillin-resistant *Staphylococcus aureus* (MRSA) in trypticase soy broth containing sponge material from 5 CareDry female external urine wicking devices after passage of 0, 0.1, 1, 2, or 3 liters of artificial urine through the device. The control was trypticase soy broth with no sponge material. Error bars show standard error.

Figure 2 shows the growth curve for MRSA in the presence of sections of the CareDry FEUWD after differing amounts of artificial urine had flowed through the device. For all tested sections, growth of MRSA was suppressed by >4 log_10_ CFU by 24 hours of incubation (*P* <.001).

## Discussion

We found that sponge sections from a FEUWD impregnated with the preservatives thiabendazole and zinc pyrithione were effective in preventing growth of several bacterial and fungal pathogens in broth culture, whereas 3 other FEUWDs did not. The inhibitory activity persisted after passage of 3 liters of artificial urine through the device. Zinc pyrithione exhibited in vitro activity against the test microbes, whereas thiabendazole did not. These results suggest that the FEUWD impregnated with zinc pyrithione could potentially reduce overgrowth of microbes within and in proximity to the device.

Our finding that zinc pyrithione has substantial antimicrobial activity against bacterial pathogens and *Candida* species is consistent with previous studies.^2^ Blanchard et al.^9^ reported similar activity of zinc pyrithione against bacterial pathogens and demonstrated antibiofilm activity. Pimentel et al.^10^ demonstrated that zinc pyrithione has activity against *C. auris*, with synergistic activity in combination with nystatin. Our finding of persistent activity after passage of urine through the device impregnated with zinc pyrithione is consistent with prior reports of persistent activity when incorporated into cellulose sponges or fabrics.^5,6^

The clinical significance of the antimicrobial activity of the CareDry FEUWD is uncertain. The antimicrobial activity might reduce overgrowth of urinary pathogens in proximity to the urethra and bladder, thereby reducing the risk for FEUWD-associated UTI. However, studies in patients will be needed to test this hypothesis.

This study has some limitations. The study was a laboratory evaluation and should not be interpreted as evidence of clinical benefit. A small number of pathogens were studied. MRSA was tested although it is not a common urinary pathogen. Urine of patients using FEUWDs may contain antimicrobials and other compounds that may alter the antimicrobial activity of zinc. Clinical studies will be needed to determine if antimicrobial-impregnated FEUWDs reduce the risk for overgrowth of urinary pathogens and bacteriuria and to determine if there are any adverse effects.

## Figure 1. Long description

Five line graphs depict the growth of test microbes in nutrient broth containing sponge material from female external urine wicking devices. Each graph represents a different microbe: MRSA, Escherichia coli, Klebsiella pneumoniae, Candida albicans, and Candidozyma auris. The x-axis represents time after inoculation in hours, ranging from 0 to 48 hours. The y-axis represents the log10 of colony-forming units per milliliter (CFU/mL), ranging from 0 to 10. Each graph includes five lines representing different devices: Caredry, Versette, PureWick, PrimaFit, and a control. Panel A shows the growth of MRSA, Panel B shows the growth of Escherichia coli, Panel C shows the growth of Klebsiella pneumoniae, Panel D shows the growth of Candida albicans, and Panel E shows the growth of Candidozyma auris. Each graph shows the growth trends of the microbes over time for each device. Error bars indicate the standard error.

Navigate back to Figure 1..
